# Prevalence of Undiagnosed Inflammatory Bowel Disease in Spondyloarthritis Patients

**DOI:** 10.3390/jcm14134569

**Published:** 2025-06-27

**Authors:** Jesús Sanz-Sanz, Ana Gutiérrez-Casbas, Zulema Plaza, Jordi Gratacós, Iago Rodríguez-Lago, Ignacio Marín-Jiménez, Elisa Trujillo-Martín, Eva Pérez-Pampín, Manuel Barreiro-de Acosta, María Vanesa Hernández-Hernández, Marta Carrillo-Palau, María Luz García-Vivar, María Carmen Muñoz-Villafranca, Maria Lourdes Ladehesa-Pineda, Eva Iglesias-Flores, Carolina Merino-Argumánez, Yago González-Lama, Marta Arévalo-Salaet, Xavier Calvet, Federico Díaz-Gonzalez

**Affiliations:** 1 Departamento de Reumatología, Hospital Universitario Puerta de Hierro, Majadahonda, 28222 Madrid, Spain; jesussanzsanz4@gmail.com (J.S.-S.); merinocarol11@gmail.com (C.M.-A.); 2 Departamento de Gastroenterología, Hospital General Universitario de Dr. Balmis de Alicante Servicio Digestivo, ISABIAL y CIBERehd, 03010 Alicante, Spain; gutierrez_anacas@gva.es; 3 Unidad de Investigación, Sociedad Española de Reumatología, 28001 Madrid, Spain; zulema.plaza@ser.es; 4 Servicio de Reumatología, Parc Taulí, Hospital Universitari, Institut d’Investigació i Innovació Parc Taulí (I3PT-CERCA), Universitat Autònoma de Barcelona, 08208 Sabadell, Spain; jgratacosmas@gmail.com (J.G.); arevalo.parctauli@gmail.com (M.A.-S.); 5 Departamento de Gastroenterología, Hospital Universitario Galdakao-Usansolo, Instituto de Investigación Sanitaria Biobizkaia, 48960 Galdakao, Spain; 6 Servicio Aparato Digestivo, Hospital General Universitario Gregorio Marañón, Instituto de Investigación Sanitaria Gregorio Marañón (IiSGM), 28007 Madrid, Spain; drnachomarin@hotmail.com; 7 Departamento de Reumatología, Hospital Universitario Canarias, Calle Ofra s/n, 38320 La Laguna, Spain; elisatm@telefonica.net (E.T.-M.); mvhhernandez@gmail.com (M.V.H.-H.); 8 Departamento de Reumatología, Instituto de Investigación Sanitaria de Santiago (IDIS), Hospital Clínico Universitario de Santiago de Compostela, 15706 Santiago de Compostela, Spain; evappampin@gmail.com; 9 Unidad de Enfermedad Inflamatoria Intestinal, Departamento de Gastroenterología, Hospital Clínico Universitario de Santiago de Compostela, 15706 Santiago de Compostela, Spain; manubarreiro@hotmail.com; 10 Departamento de Gastroenterología, Hospital Universitario Canarias, 38320 La Laguna, Spain; martacarry@yahoo.es; 11 Departamento de Reumatología, Hospital Universitario Basurto, 48013 Bilbao, Spain; marialuz.garciavivar@osakidetza.eus; 12 Departamento de Gastroenterología, Hospital Universitario Basurto, 48013 Basurto, Spain; mariadelcarmen.munozvillafranca@osakidetza.eus; 13 Departamento de Reumatología, Hospital Reina Sofia, 14004 Córdoba, Spain; lourdesladehesapineda@gmail.com; 14 Departamento de Gastroenterología, Hospital Reina Sofia, 14004 Córdoba, Spain; evaiflores@gmail.com; 15 Departamento de Gastroenterología, Hospital Universitario Puerta de Hierro, Majadahonda, 28222 Madrid, Spain; ygonzalezlama@gmail.com; 16 Servei d’Aparell Digestiu, Parc Taulí, Hospital Universitari, Institut d’Investigació i Innovació Parc Taulí (I3PT-CERCA), Universitat Autònoma de Barcelona, 08208 Sabadell, Spain; xavier.calvet.c@gmail.com; 17 Centro de Investigación Biomédica En Red de Enfermedades Hepáticas y Digestivas (CIBERehd), Instituto de Salud Carlos III, 28029 Madrid, Spain; 18 Departamento de Medicina Interna, Dermatología y Psiquiatría, Universidad de La Laguna, 38320 La Laguna, Spain; 19 Instituto Universitario de Tecnologías Biomédicas (ITB), Universidad de La Laguna, 38320 La Laguna, Spain

**Keywords:** prevalence undiagnostic, inflammatory bowel disease, fecal calprotectin, axial spondylarthritis, psoriatic arthritis

## Abstract

**Background/Objectives**: The prevalence of inflammatory bowel disease (IBD) in spondyloarthritis (SpA) patients is unknown. Our objective was to assess the prevalence of undiagnosed IBD in SpA patients, including those with axial spondylarthritis (axSpA) or psoriatic arthritis (PsA). Additionally, we examined fecal calprotectin (FC) levels in relation to the accuracy of IBD diagnosis. **Methods**: EISER was a cross-sectional, multicenter, observational, rheumatologist–gastroenterologist collaborative study. Patients with SpA naïve to biologics were recruited. Demographic and clinical characteristics, disease activity, and treatment information were collected. Patients with FC ≥ 80 µg/g or IBD-related symptoms underwent a colonoscopy or video capsule endoscopy. Receiver operating characteristic analysis assessed the predictive value of FC for IBD diagnosis. **Results**: Of the 570 patients recruited, 494 were evaluable for the main outcome, 248 (50.2%) had axSpA, and 246 (49.8%) had PsA. Overall, 28/494 patients were diagnosed with IBD (5.7%, 95%CI 3.6–7.7). Sorted by clinical entity, 22 (8.9%, 95%CI 5.3–12.4) axSpA and 6 (2.4%, 95%CI 0.5–4.4) PsA patients had a diagnosis of IBD: 24 (86%, 95%CI 79.4–92.6) had ileal/ileocolonic Crohn’s disease (CD), 3 (11%, 95%CI 5.1–16.9) unclassified IBD, and 1 (3.5%, 95%CI 0.0–6.9) ulcerative colitis. The ROC curve for FC and IBD diagnosis (AUC: 0.870, *p* < 0.001, 95%CI 83.7–89.8) showed that an FC ≥ 147 µg/g had a positive predictive value of 17.4% (95%CI 14.5–20.8) **Conclusions**: In SpA, the prevalence of undiagnosed IBD was 5.7%, higher in axSpA (8.9%) than in PsA (2.4%) patients, with CD being the most common. SpA patients with FC levels < 147 µg/g had a very low probability of IBD.

## 1. Introduction

Spondyloarthritis (SpA) comprises a group of interrelated rheumatic diseases including ankylosing spondylitis (AS), psoriatic arthritis (PsA), inflammatory bowel disease (IBD)-associated arthritis, and reactive arthritis, which can be categorized depending on its clinical presentation as axial (ax)SpA (including radiographic, r-axSpA, and non-radiographic, nr-axSpA) or peripheral SpA [1]. In addition to arthritis, enthesitis, and/or dactylitis, patients with SpA may also present extra-musculoskeletal manifestations, with uveitis, psoriasis, and IBD being the most frequently diagnosed [2,3].

The prevalence of IBD has been reported to be as high as 6.8% in patients with AS [2] and 3.3% in those with PsA [3]. However, the proportion of biologic-naïve SpA patients with undiagnosed IBD remains unknown. The occurrence of IBD in patients with AS is associated with higher disease activity, poorer physical functioning, poorer patient global well-being, greater healthcare resource utilization, more IBD-related procedures, and higher glucocorticoid use, resulting in increased healthcare costs [4]. Given the worsening prognosis and therapeutic implications in patients with SpA and concomitant IBD, early diagnosis of IBD through coordination between rheumatologists and gastroenterologists is crucial [5]. Although some questionnaires [6] or diagnostic algorithms [5] have been proposed to help rheumatologists better screen for IBD in patients with SpA, their effectiveness in clinical practice has barely been investigated [7].

Fecal calprotectin (FC) is a non-invasive biomarker of intestinal inflammation [8]. Although the levels in fecal samples can be influenced by the use of non-steroidal anti-inflammatory drugs (NSAID) or proton pump inhibitors (PPI) [9], FC is often used as tool to differentiate between inflammatory and non-inflammatory gastrointestinal disorders. A recent systematic review concluded that FC may be useful for screening patients with rheumatic diseases who may require a colonoscopy to exclude a diagnosis of IBD [10].

The primary objective of this study was to estimate the prevalence of undiagnosed IBD in SpA patients. The levels of FC associated with the diagnosis of IBD in this patient group was also evaluated.

## 2. Materials and Methods

### 2.1. Study Design

The EISER project was a multicenter, cross-sectional study conducted in the rheumatology and gastroenterology departments of 13 hospitals, in the context of a collaboration between the Spanish Society of Rheumatology (SER) and the Spanish Working Group on Crohn’s Disease and Ulcerative Colitis (GETECCU). This study was approved by the Medical Research Ethics Committee of the Hospital Puerta de Hierro Majadahonda (Madrid, Spain, reference FER-PRE-2020-01-H.U.P.H:108/20). This study was conducted in accordance with the principles of the Declaration of Helsinki and all participants provided written informed consent before enrolling in this study.

### 2.2. Selection Criteria

The patients included were diagnosed with PsA or axSpA in accordance with the Classification for Psoriatic Arthritis Criteria [11] and The Assessment of Spondylarthritis international Society Criteria [12], respectively. All patients were under regular follow-up by rheumatology services at the participating centers. Patients with a previous diagnosis of any other rheumatic disease or chronic gastrointestinal disease, including IBD, were excluded from this study. The colorectal cancer screening program in Spain targets individuals over 50 years old. Our study only included participants over 50 years old who had not undergone a complete colonoscopy in the past three years or those who had a colonoscopy that did not meet the minimum quality standards. Colonoscopies were considered valid and reliable if they reached the cecum and achieved adequate bowel cleansing according to the Boston Bowel Preparation Scale [13]. Because some biologics used in SpA patients may also affect intestinal inflammation (e.g., anti-TNF), while others have been associated with disease exacerbation (e.g., anti-IL-17) [14] that could lead to an under- or overestimation of results, patients who were under active biologic therapy were excluded. Due to its anti-inflammatory effect on the intestinal mucosa, patients who had taken prednisone > 10 mg/day for any reason in the 30 days prior to enrollment were also excluded.

### 2.3. Study Procedures and Assessments

From December 2020 through July 2022, all patients diagnosed with PsA or AS who were in regular outpatient follow-up and met the selection criteria were invited to participate in this study. Information on demographics and lifestyle habits was collected. The following variables were recorded by the rheumatologist: diagnosis, date of diagnosis, disease activity, current treatment, the Patient Global Assessment of disease activity using a numerical scale rating of 0–10, the Bath Ankylosing Spondylitis Disease Activity Index (BASDAI) [15] and the Ankylosing Spondylitis Disease Activity Score (ASDAS) [16] for axSpA patients; Disease Activity in Psoriatic Arthritis (DAPSA) [17] for PsA patients; and the red-flag symptoms for the screening of IBD in patients with SpA as described by the Inflammatory Bowel and Joint Pathology working group PIIASER [5] (Supplementary Table S1). Rheumatologists also requested FC from all patients. A Quantum Blue^®^fCAL rapid test (BÜHLMANN Laboratories AG, Shönenbuch, Switzerland) was used to determine FC levels. The gastroenterologist to whom the patient was referred subsequently reviewed the FC test results along with the relevant clinical information. According to the algorithm described by the manufacturer, a cut-off point ≥ 80 µg/g in non-NSAID users and ≥160 µg/g in patients taking NSAIDs was used to indicate colonoscopy. Supplementary Figure S1 shows the flowchart of patients according to baseline FC levels, symptoms, and NSAID use.

In accordance with protocol (based on FC values) or clinical suspicion, a colonoscopy was performed to rule out previously undiagnosed IBD. If the colonoscopy was negative, a video capsule endoscopy (VCE) was carried out. The findings were assessed using the Simple Endoscopic Score (SES-CD) for evaluating Crohn’s Disease (CD) endoscopic severity [18], or the Ulcerative Colitis Endoscopic Index of Severity (UCEIS) for ulcerative colitis (UC) [19]. In mucosal biopsies obtained during the colonoscopy, a diagnosis of IBD was confirmed through pathological analysis [20]. If VCE was contraindicated using a Patency Agile capsule (Given Imaging Ltd., Yoqneam, Israel), a magnetic resonance enterography (MRE) was then conducted per protocol. VCE findings were scored using the Lewis index [21], and CD was diagnosed according to Tukey M. et al. [22].

### 2.4. Statistical Analysis

The sample size calculation, which was used to estimate an IBD prevalence of 10% (Stebbings et al. [6] reported a CD prevalence of 7.8%) with an absolute precision of 2.5% and a confidence level of 95% yielded a sample of 540 patients. Quantitative variables were described using mean and standard deviation (SD) or median and interquartile range (IQR). Qualitative variables were described with absolute and relative frequencies. Cohen’s kappa coefficient was used to assess the agreement between rheumatologists and gastroenterologists on the criteria for IBD screening [5]. Multiple logistic regression analysis was used to analyze the association between demographics, clinical characteristics, and treatments with the presence of an FC level ≥ 80 µg/g (yes/no), including all those variables with *p* < 0.2 in the univariable analysis. The Wald forward method was used to identify factors associated with FC ≥ 80 µg/g.

Logistic regression was used to identify factors associated with IBD diagnosis. The predictive ability of the FC level for a diagnosis of IBD was determined using receiver operating characteristic (ROC) analysis and by calculating the area under the curve (AUC) as an overall summary of diagnostic accuracy. Youden’s index was used to determine the best cut-off point. To compare the different variables, a Student’s *t*-test, U-Mann–Whitney test, Fisher’s exact test, or Pearson’s chi-square was used as indicated.

All analyses were performed using SPSS version 26.0 (IBM SPSS Statistics; IBM Corp., Armonk, NY, USA) and MedCalc version 22 (MedCalc Software, Ostend, Belgium). A *p* value < 0.05 was considered significant.

## 3. Results

### 3.1. Patients’ Disposition and Characteristics

**Figure 1:**
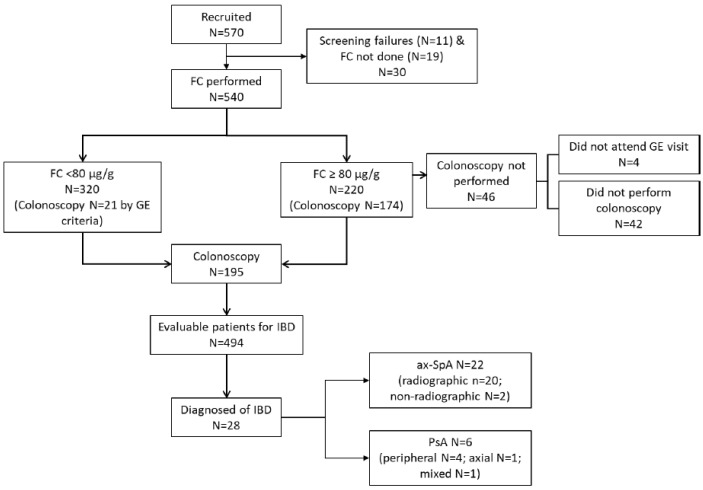
Flowchart of patients included in this study. FC: fecal calprotectin, IBD = inflammatory bowel disease, GE = gastroenterologist, axSpA: axial spondylarthritis, PsA: psoriatic arthritis.

Of the 570 patients recruited, 30 were excluded (Figure 1). In the final analysis 540 patients were included: 273 (50.6%) had axSpA (198 r-axSpA;75 nr-axSpA) and 267 (49.4%) had PsA (17 axial, 196 peripherals, and 54 mixed).

Table 1 shows the demographic and clinical characteristics, as well as the treatments undergone by patients. The mean disease activity of the enrolled population was low (DAPSA: 10.4 ± 8.3) in PsA and moderate to high (BASDAI: 3.6 ± 2.3, ASDAS-ESR: 2.4 ± 1.0) in axSpA patients. AxSpA vs. PsA patients received more NSAIDs (69.2% vs. 39.0%) and PPIs (38.8 vs. 34.5), and fewer disease-modifying antirheumatic drugs (DMARDs) (11.4% vs. 71.5%).

### 3.2. Fecal Calprotectin Levels

To evaluate the global impact of NSAID and PPI use on FC levels in patients with SpA, we assessed FC levels and the percentage of patients with FC ≥ 80 µg/g in the total population and by clinical entity. This evaluation encompassed those patients taking NSAIDs, PPIs, both, or neither (Table 2). FC was tested in 540 patients, of whom 220 (40.7%) showed FC ≥ 80 µg/g. The median (IQR) level of FC was 47.0 µg/g (30.0–169.0) for PsA patients, 66.0 µg/g (30.0–222.5) for axSpA patients (Table 2), 73.0 µg/g (30.7–249.0) for radiographic patients, and 41.0 µg/g (30.0–118.0) for nr-axSpA patients. PPI and PPI plus NSAID treatments were associated with a significant increase in both FC levels and the percentage of patients with FC ≥ 80 µg/g, with no differences between axSpA and PsA patients. However, NSAID treatment has no significant effect on FC levels in those with SpA.

Univariable and multivariable logistic regression analyses that evaluated the association between demographic and clinical characteristics and treatments involving the presence of an FC level > 80 µg/g are shown in Table 3. The multivariate logistic regression analysis showed that the factors associated with a greater likelihood of presenting an FC ≥ 80 µg/g were disease duration (OR 1.026, 95%CI 1.008–1.044, *p* = 0.004), the use of DMARD(s) (OR 1.522, 95%CI 1.016–2.281, *p* = 0.042), and the use of a PPI (OR 5.152, 95%CI 3.454–7.683, *p* < 0.001).

### 3.3. Prevalence of Inflammatory Bowel Disease

Of the 540 patients included, 494 were evaluable for this study’s primary objective, assessing previously undiagnosed IBD in patients with SpA: 248 with axSpA and 246 with PsA (Figure 1). A total of 174 patients with an FC ≥ 80 µg/g and 21 with an FC < 80 µg/g underwent a colonoscopy (see Figure 1). A total of 149 patients had a valid colonoscopy: 68 out of 82 (82.9%) were PsA patients, and 81 out of 92 (88.0%) were axSpA patients. Among them, 59 patients (39.6%) had abnormal findings, mostly among axSpA patients (N = 39, 66.1%), compared to PsA patients (N = 20, 33.8%) (*p* = 0.028). Overall, 28/494 patients with SpA were diagnosed with IBD (5.7%, 95%CI 3.6–7.7). By clinical entity, 22/248 patients with axSpA had a diagnosis of IBD (8.9%, 95%CI 5.3–12.4) as did 6/246 patients with PsA (2.4%, 95%CI 0.5–4.4). Among patients with axSpA, the prevalence of IBD was significantly higher (*p* = 0.046) in r-axSpA patients, at 20/179 (11.2%, 95%CI 6.5–15.8), than in nr-axSpA patients, at 2/69 (2.9%, 95%CI 0.01–6.9). In the contingency analysis, no association was found between sex and IBD diagnosis in either axSpA or PsA patients.

The key characteristics of the 28 patients with IBD are shown in Table 4. Twenty-four of them had a diagnosis compatible with CD (86%, 95%CI 79.4–92.6), three had unclassified IBD (11%, 95%CI 5.1–16.9), and one had a diagnosis of UC (3.5%, 95%CI 0.0–6.9). In four patients, the diagnosis of IBD was made by VCE. In eight patients who had a positive colonoscopy, VCE was also performed to determine the proximal extent of the lesions according to the gastroenterologist’s criteria. No patients underwent an MRE.

A summary of colonoscopy and VCE findings in patients diagnosed with IBD is presented in Supplementary Table S2. Seven (25.0%) patients with IBD also presented suggestive symptoms. In the bivariate analysis (Supplementary Table S3), factors significantly associated with a diagnosis of IBD were a diagnosis of axSpA vs. PsA (OR 3.89; 95%CI 1.55–9.78, *p* = 0.002), gastrointestinal symptoms suggestive of IBD (OR 2.77; 95%CI 1.12–6.85, *p* = 0.032), chronic abdominal pain (OR 3.87; 95%CI 1.05–14.36, *p* = 0.030), a vitamin B12 deficiency (OR 3.36; 95%CI 1.07–10.54, *p* = 0.028), an FC ≥ 80 µg/g (OR 27.93; 95%CI 6.54–119.2, *p* < 0.001), and positive HLAB27 (OR 4.39; 95%CI 1.47–13.09, *p* = 0.004). As previously mentioned, 21 patients with CF < 80 µg/g underwent a colonoscopy based on a gastroenterologist’s clinical suspicion, according to the criteria shown in Supplementary Table S1. Of these patients, two (9.5%) were finally diagnosed with IBD. IBD-related criteria (Supplementary Table S1) assessed independently by gastroenterologists and rheumatologists showed a concordance of 78.6% (kappa = 0.25, *p* < 0.001). The symptoms consistent with IBD, both by disease type and in the whole population, are shown in Supplementary Table S4.

### 3.4. Diagnostic Performance of Fecal Calprotectin

**Figure 2:**
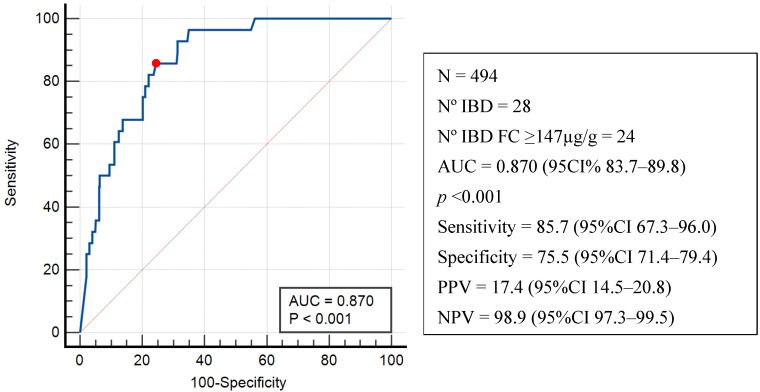
Receiver operating characteristic curve (ROC) for the prediction of IBD based on basal FC levels in the evaluable population for IBD. The frame shows the number of patients analyzed (N), the number of patients with IBD (N IBD), and the number with IBD and FC ≥ 147 µg/g (N IBD FC ≥ 147). Also shown are the corresponding area under the curve (AUC), the *p*-values, and the positive (PPV) and the negative (NPV) predictive values. Red dot shows the Youden index.

Figure 2 shows the ROC curve for FC levels and an IBD diagnosis in the 494 evaluable patients with an AUC of 0.870 (95%CI 83.7–89.8, *p* < 0.001). According to the Youden index, the highest sensitivity (85.7%) and specificity (75.5%) corresponded to an FC level ≥ 147 µg/g, with a positive predictive value (PPV) of 17.4% (95%CI 14.5–20.8) and a negative predictive value (NPV) of 98.9% (95%CI 97.3–99.5). When HLA-B27 positivity, SpA type, and symptoms compatible with IBD diagnosis were included in the analysis, no difference was observed in the ROC curve for IBD diagnosis (AUC: 0.876; 95%CI: 83.8–90.7).

To determine whether the use of NSAIDs and/or PPIs resulted in an overestimation of the Youden index, ROC curves were analyzed separately, excluding these three patient groups. Supplementary Figure S2 shows ROC curves for patients not taking an NSAID(A), PPI(B), or either(C). In these analyses, AUCs measured 0.846 (95%CI 0.833–0.898, *p* < 0.001), 0.892 (95%CI 0.853–0.924), and 0.884 (95%CI 0.839–0.921), respectively, which proved similar to those obtained in the total population analysis, maintaining the Youden index at FC ≥ 147 µg/g.

## 4. Discussion

Our study showed that the prevalence of undiagnosed IBD in SpA patients is 5.7%, and higher among those with axSpA (8.9%), but this was also relevant to the PsA population (2.4%), with most of them involving endoscopic and histopathological findings of CD.

The prevalence of diagnosed IBD in axSpA patients has been described in a meta-analysis, as well as in a study based on administrative data ranging from 6.8% [2] to 8.5% [23]. CD was the most common diagnosis in the database study [23]. Recently, however, a higher prevalence of IBD has been described in axSpA patients: 11.8%, most of whom were diagnosed with UC (90%) [24]. With respect to PsA patients, Bergman et al. [23] reported a prevalence of IBD in 3.8% among the 22,205 enrolled patients, most corresponding to CD (60%), while a single-center study in Spain showed a prevalence of 3.3% among 306 patients [25]. It is important to note that in our study most patients with PsA exhibited peripheral disease, with IBD proving more common in its axial than peripheral form [26].

We found that the prevalence of undiagnosed IBD was significantly higher in r-axSpA than in nr-axSpA patients (11.2% vs. 2.9%), a finding consistent with previous results [27]. Although there seems to be agreement that nr-axSpA truly represents either an early stage or an abortive type of the radiographic form [28], our data suggest that they differ, at least in terms of intestinal involvement.

In our study, only 7 of 28 patients with IBD reported symptoms consistent with these disorders. This finding could be interpreted in the context that SpA patients may be prone to oligosymptomatic or ‘silent’ forms of IBD [29,30]. Evidence has shown that a minority of SpA patients with subclinical gastrointestinal inflammation may develop IBD over time, with a higher prevalence of CD. Cohort studies and meta-analyses demonstrate a lifetime risk of IBD in SpA patients ranging from 4 to 7% [2,31,32]. In this regard, a population-based matched cohort study of 4101 axSpA patients found that 4% had a pre-existing diagnosis of IBD. The diagnosis of IBD increased to 7.5% at the end of the 20-year follow-up [33]. The prospective follow-up of patients with IBD in our series may clarify the likelihood of these patients developing symptomatic IBD in the future. Awareness of the disease and being under regular follow-up may also explain the low rate of symptoms observed in our cohort.

The use of IBD screening criteria [5] showed a low level of concordance between rheumatologists and gastroenterologists (Cohen’s kappa index of 0.25). This lack of agreement suggests that rheumatologists may not be adequately considering gastrointestinal symptoms when assessing patients with SpA. This is an important issue because delayed diagnosis of IBD is associated with worse clinical outcomes, including an increased likelihood of stenosing and penetrating phenotypes among CD patients, as well as bowel surgery in both CD and UC patients [34].

Patients with SpA often use NSAIDs, which can damage the intestinal mucosa. Clinical history, symptoms, endoscopic findings, biopsy results, laboratory tests, and VCE imaging [21,22,35] can all help differentiate IBD from NSAID enteropathy. In our study, 12 of the 28 patients diagnosed with IBD required VCE (4 of whom had no abnormalities on colonoscopy). This is consistent with previous findings in which VCE revealed small bowel inflammation consistent with CD in 42.2% of patients with SpA, with a significant incremental yield over colonoscopy totaling 31% [29]. This suggests that when IBD is suspected in SpA patients with a normal colonoscopy, VCE should be considered.

FC is a marker of intestinal inflammation [8]. We found that 41% of SpA patients had an FC level ≥ 80 µg/g, similar to previously described cohorts [10]. Our findings suggest that longer disease duration, DMARD and PPI use, but not disease activity or NSAID use, increase the likelihood of presenting FC levels ≥ 80 µg/g. Concerning the relationship between disease duration and FC levels in patients with SpA, inconsistent results have been reported [36,37]. Although the use of NSAID(s) or PPI has been associated with a significant increase in FC levels [9,38], in our study patients with SpA treated with a NSAID did not exhibit significantly elevated FC levels, a finding that may have clinical implications.

Recently, the usefulness of FC for screening IBD in patients with rheumatic diseases has gained increasing advocacy [10]. We found that the AUC of the ROC curve for FC in diagnosing IBD was 0.870. Moreover, FC levels over 147 µg/g showed a sensitivity of 85.7% and a specificity of 75.5%, with a positive predictive value of 17.4% and a negative predictive of 98.9%. In our study, the addition of symptom screening consistent with IBD did not improve the diagnostic performance of FC. Importantly, the exclusion of patients who were receiving an NSAID, PPI, or both yielded almost identical results in the ROC analysis and the same maximum potential effectiveness of FC levels (Youden index) at ≥147 µg/g. This suggests that it may not be necessary to discontinue NSAID or PPI treatment when using FC as a screening tool for IBD in these patients. On the other hand, adding IBD symptoms to our predictive model did not improve the diagnostic performance of FC, indicating the necessity of this biomarker for selecting patients for further diagnostic tests.

A limitation of our study was that colonoscopies were not performed in 21% of patients with elevated FC levels, which certainly contributed to the widening of the result confidence intervals. As all FC values were determined using the Quantum Blue^®^ assay, other FC assay systems may have different absolute cut-off values. The exclusion of patients treated with any biologics or prednisone > 10 mg/day carries the advantage of avoiding the anti-inflammatory effects of these agents on the intestinal mucosa, thus providing a better idea of the true prevalence of undiagnosed IBD in SpA patients. However, the exclusion of biologics may result in the loss of patients with more aggressive disease and potentially greater bowel involvement. Another possible limitation is that, for ethical reasons, patients did not discontinue their conventional background treatments, including the 60% of those on methotrexate and the 13% on sulfasalazine, compounds that may have potentially reduced intestinal inflammation. Among the strengths of this study are that it involved close coordination between rheumatologists and gastroenterologists, it included many patients, and it used the same FC determination assay in all cases. Furthermore, it combined clinical, endoscopic VCE, and histologic assessments.

## 5. Conclusions

In summary, our results show a prevalence of undiagnosed IBD in those SpA patients who do not take biologics, some 5.7%, higher in those with axSpA (8.9%) than in PsA patients (2.4%), with most cases corresponding to CD. A high proportion of SpA patients have elevated FC levels. FC levels < 147 µg/g, as determined by the Quantum Blue^®^ assay, indicate a low probability of an IBD diagnosis in patients with SpA, regardless of NSAID and/or PPI use. In SpA, particularly in axSpA with elevated FC levels and normal colonoscopy results, VCE should be considered to investigate the possibility of small bowel CD.

## Figures and Tables

**Table 1:** Demographics, clinical characteristics, and treatments of the included population.

Characteristics	axSpA N = 273	PsA N = 267
Sex (women), n (%)	123 (45.1)	143 (53.6)
Age (years), mean ± SD	50.1 ± 12.6	54.5 ± 12.7
Age at diagnosis (years), mean ± SD	35.6 ± 12.1	44.9 ± 12.7
Disease duration (years), median (IQR)	14.2 (3.7; 22.4)	7.5 (2.3; 14.3)
Smoking, n (%)		
Never smoker	126 (46.2)	135 (50.6)
Former smoker	98 (35.9)	84 (31.5)
Current smoker	49 (17.9)	48 (17.9)
Alcohol use (yes), n (%)	128 (46.9)	93 (34.8)
Characteristics	axSpA N = 273	PsA N = 267
HLA-B27, positive/evaluable (%)	195/240 (81.3)	32/171 (18.7)
Disease activity		
BASDAI, mean ± SD	3.6 ± 2.3	3.6 ± 2.4
DAPSA, mean ± SD	-	10.4 ± 8.3
ASDAS_CRP, mean ± SD	2.3 ± 1.0	2.2 ± 1.1
ASDAS_ESR, mean ± SD	2.4 ± 1.0	2.4 ± 1.1
Patient Global Assessment, mean ± SD	4.4 ± 2.7	3.9 ± 2.7
Treatments		
NSAID, n (%)	189 (69.2)	104 (39.0)
DMARD, n (%)	31 (11.4)	191 (71.5)
Methotrexate	11 (4.0)	147 (55.1)
Leflunomide	2 (0.7)	24 (9.0)
Sulfasalazine	20 (7.3)	13 (4.9)
Apremilast	-	10 (3.7)
PPI, n (%)	106 (38.8)	92 (34.5)

Abbreviations: ASDAS_CRP: Ankylosing Spondylitis Disease Activity Score with CRP; ASDAS_ESR: Ankylosing Spondylitis Disease Activity Score with ESR; BASDAI: Bath Ankylosing Spondylitis Disease Activity Index; DAPSA: Disease Activity in PSoriatic Arthritis; DMARD: disease-modifying antirheumatic drug; ESR: erythrocyte sedimentation rate; IQR: interquartile range; NSAID: non-steroidal anti-inflammatory drug; PPI: proton pump inhibitors; SD, standard deviation; VAS: visual analog scale.

**Table 2:** Fecal calprotectin levels and number of patients with fecal calprotectin > 80 µg/g in the entire population and by clinical entity, considering separately those patients not taking NSAIDs, PPIs, or either.

	All Patients		Excluding NSAID Users		Excluding PPI Users		Excluding NSAID + PPI Users	
	FC N; FC µg/g (IQR)	FC ≥ 80 µg/g N (%)	FC N; FC µg/g (IQR); *p*-Value	FC ≥ 80 µg/g N (%); *p*-Value	FC N; FC µg/g (IQR); *p*-Value	FC ≥ 80 µg/g N (%); *p*-Value	FC N; FC µg/g (IQR); *p*-Value	FC ≥ 80 µg/g N (%); *p*-Value
**Overall**	540; 56.5 (30.0–192.0)	220 (40.7)	414; 53.5 (30.0–157.5); 0.502	166 (40.1); 0.894	342; 40.0 (30.0–94.3); <0.001	92 (26.9); <0.001	277; 39.0 (30.0–93.5); <0.001	75 (27.1); <0.001
**axSpA**	273; 66.0 (30.0–222.5)	117 (42.9)	187; 60.0 (30.0–171.0); 0.582	78 (41.7); 0.807	167; 46.0 (30.0–123.0); 0.029	53 (31.7); 0.021	122; 46.0 (30.0–117.3); 0.032	39 (32.0); 0.045
**PsA**	267; 47.0 (30.0–169.0)	103 (38.6)	227; 49.0 (30.0–155.0); 0.785	88 (38.8); 0.966	175; 35.0 (30.0–71.0); 0.001	39 (22.3); <0.001	155; 35.0 (30.0–75.0); 0.001	36 (23.2); 0.001

Data are expressed as N, median (IQR), *p* value, and as number of patients and (%). The *p* value represents the statistical significance of the FC values and the number of patients with FC > 80 µg/g in each box with respect to their values in the all-patients column by row, using the U-Mann–Whitney test and Fisher’s exact test, respectively. Abbreviations: axSpA: axial spondylarthritis; FC: fecal calprotectin; IQR: interquartile range; N: number of patients; NSAID: non-steroidal anti-inflammatory drug; PPI: proton pump inhibitor; PsA: psoriatic arthritis.

**Table 3:** Logistic regression analysis evaluating the association between demographics, clinical characteristics, and treatments in patients with fecal calprotectin levels ≥ 80 µg/g.

Variables	Univariable Odds Ratio (IC_95%_); *p*-Value	Multivariable Odds Ratio (IC_95%_); *p*-Value
Diagnostic EspAax (ref. PsA)	1.194 (0.847–1.684); 0.312	
Radiographic	1.439 (0.992–2.088); 0.055	
Not radiographic	0.704 (0.407–1.220); 0.211	
Disease duration (years)	1.023 (1.008–1.039); 0.002	1.026 (1.008–1.044); 0.004
Patient global assessment	1.054 (0.988–1.124); 0.109	
EQ-5D-5L (VAS) visual analog scale	0.984 (0.975–0.993); <0.001	
Laboratory values		
CRP	0.998 (0.977–1.020); 0.873	
ESR	1.015 (1.001–1.030); 0.033	
HLAB27 positive	1.203 (0.810–1.787); 0.360	
Disease activity		
TJC68	0.978 (0.908–1.054); 0.559	
SJC66	0.991 (0.891–1.102); 0.866	
ASDAS-CRP	1.100 (0.898–1.348); 0.356	
BASDAI	1.007 (0.920–1.102); 0.882	
ASDAS-ESR	1.212 (0.981–1.497); 0.075	
Treatments		
NSAID	1.085 (0.768–1.531); 0.644	
DMARD	1.310 (0.925–1.856); 0.128	1.522 (1.016–2.281); 0.042
PPI	4.969 (3.409–7.243); <0.001	5.152 (3.454–7.683); <0.001

Abbreviations: ASDAS: Ankylosing Spondylitis Disease Activity Score; BASDAI: Bath Ankylosing Spondylitis Disease Activity Index; CRP: C-reactive protein; DMARD: disease-modifying antirheumatic drug; ESR: erythrocyte sedimentation rate; NSAID: non-steroidal anti-inflammatory drug; PPI: proton pump inhibitors; SJC, swollen joint count; TJC: tender joint count; VAS: visual analog scale.

**Table 4:** Characteristics of patients with a diagnosis of IBD.

Type of SpA	Sex	Age (Years)	FC (µg/g)	CRP (mg/L)	Family History of IBD	IBD Symptoms	Colonoscopy Findings	Histology Findings	VCE	VCE Findings	IBD DX
r-axSpA	F	46	38	4.10	N	Y	N	-	Y	Y	CD
r-axSpA	M	54	88	0.80	N	N	Y	Not done	Y	Y	CD
nr-axSpA	F	20	90	3.70	N	N	N	-	Y	Y	CD
r-axSpA	M	42	148	6.00	N	N	Y	CII	N	-	CD
r-axSpA	M	54	177	3.15	Y	N	Y	CII	Y	Y	CD
r-axSpA	M	48	186	0.75	N	N	Y	CII	N	-	CD
r-axSpA	F	75	265	0.40	Y	N	Y	CII	N	-	CD
r-axSpA	M	53	285	3.86	N	Y	N	-	Y	Y	CD
r-axSpA	M	52	306	22.30	N	N	Y	CII	Y	Y	CD
nr-axSpA	F	27	306	4.40	N	N	Y	Cryptitis	N	-	UC
r-axSpA	M	43	360	5.70	N	Y	N	-	Y	Y	CD
r-axSpA	F	56	464	0.98	N	N	Y	CII, Erosion/ulceration	N	-	Unclassified
r-axSpA	M	52	500	6.70	N	N	Y	Erosion/ulceration	Y	Y	CD
r-axSpA	M	54	547	0.40	Y	N	Y	Not done	Y	Y	CD
r-axSpA	M	48	664	9.59	N	Y	Y	CII and AD	N	-	CD
r-axSpA	M	69	958	0.12	N	N	Y	AD	N	-	CD
r-axSpA	F	51	974	10.40	N	N	Y	AD	Y	Y	CD
r-axSpA	M	61	1000	7.90	N	Y	Y	Mucin depletion, AD, Erosion/ulceration, basal Plasmacytosis and AII	N	-	CD
r-axSpA	M	68	1000	12.90	Y	N	Y	Not done	Y	Y	CD
r-axSpA	M	73	1000	4.10	N	N	Y	AII and CII	N	-	CD
r-axSpA	M	44	1000	10.20	N	N	Y	AD	N	-	CD
r-axSpA	F	48	1000	31.80	N	Y	Y	Erosion/ulceration, AII and CII	N	-	CD
peripheralPsA	F	50	72	3.60	N	Y	Y	Granulomas, Erosion/ulceration	N	-	CD
Mixed PsA	M	61	166	2.00	N	N	Y	Not done	N	-	Unclassified
peripheralPsA	M	54	186	35.00	N	N	Y	CII	N	-	CD
Axial PsA	F	64	467	5.12	N	N	Y	AD and AII	N	-	CD
peripheralPsA	M	56	484	2.00	N	N	Y	Erosion/ulceration, AII and CII	N	-	Unclassified
peripheralPsA	F	30	732	1.10	N	N	Y	Not done	Y	Y	CD

Abbreviations: AD: Architectural distortion; AII: acute inflammatory infiltrate; CD: Crohn’s disease; CII: chronic inflammatory infiltrate; CRP: C-reactive protein; DX: diagnosis; F: female; FC: fecal calprotectin; IBD: inflammatory bowel disease; M: male; N: no; nr-axSpA: non-radiographic axial spondylarthritis; PsA: psoriatic arthritis; r-SpA: radiographic axial spondylarthritis; SpA: spondyloarthritis; UC: ulcerative colitis; VCE: video capsule endoscopy; Y: yes.

## Data Availability

The individual participant data on which the results presented in this article are based has been deidentified (text, tables, figures, and appendices), it will be made immediately available following the publication of this article. Investigators interested in data from this study should submit a methodologically sound request. Proposals should be addressed to proyectos@ser.es. To access the data, applicants must sign a data access agreement.

## Supplementary Materials

The following supporting information can be downloaded at https://www.mdpi.com/article/10.3390/jcm14134569/s1. Table S1: Criteria for the screening of inflammatory bowel disease in patients with spondyloarthritis; Table S2: Summary of findings in the colonoscopy and video capsule endoscopy in patients with IBD diagnosis; Table S3: Bivariate analysis of factors associated with a diagnosis of IBD; Table S4: Symptoms consistent with IBD by type of SpA. Figure S1: Flowchart according to basal determination of fecal calprotectin, use or not of NSAID, and presence or not of symptoms associated with IBD. Figure S2: Receiver operating characteristic curve (ROC) for prediction of IBD based on basal FC in the evaluable population for IBD, excluding (A) NSAID-, (B) PPI-, and (C) NSAID + PPI-taking patients.

## References

[1] Rudwaleit M., van der Heijde D., Landewé R., Akkoc N., Brandt J., Chou C.T., Dougados M., Huang F., Gu J., Kirazli Y. (2011). The Assessment of SpondyloArthritis international Society classification criteria for peripheral spondyloarthritis and for spondyloarthritis in general. Ann. Rheum. Dis..

[2] Stolwijk C., van Tubergen A., Castillo-Ortiz J.D., Boonen A. (2015). Prevalence of extra-articular manifestations in patients with ankylosing spondylitis: A systematic review and meta-analysis. Ann. Rheum. Dis..

[3] Pittam B., Gupta S., Harrison N.L., Robertson S., Hughes D.M., Zhao S.S. (2020). Prevalence of extra-articular manifestations in psoriatic arthritis: A systematic review and meta-analysis. Rheumatology.

[4] Essers I., Ramiro S., Stolwijk C., Blaauw M., Landewé R., van der Heijde D., Bosch F.V.D., Dougados M., van Tubergen A. (2014). Characteristics associated with the presence and development of extra-articular manifestations in ankylosing spondylitis: 12-year results from OASIS. Rheumatology.

[5] Sanz J.S., Roura X.J., Seoane-Mato D., Montoro M., Gomollon F., Grupo de Trabajo del proyecto PIIASER (2018). Screening of In-flammatory Bowel Disease and Spondyloarthritis for Referring Patients Between Rheumatology and Gastroenterology. Reumatol. Clin..

[6] Stebbings S., Jenks K., Treharne G.J., García J.A., Schultz M., Highton J., Dudley-Brown S. (2011). Validation of the Dudley Inflammatory Bowel Symptom Questionnaire for the assessment of bowel symptoms in axial SpA: Prevalence of clinically relevant bowel symptoms and association with disease activity. Rheumatology.

[7] Gutiérrez-Sánchez J., Parra-Izquierdo V., Flórez-Sarmiento C., Jaimes D.A., De Ávila J., Bello-Gualtero J.M., Ramos-Casallas A., Chila-Moreno L., Pacheco-Tena C., Beltrán-Ostos A. (2022). Implementation of screening criteria for inflammatory bowel disease in patients with spondyloarthritis and its association with disease and endoscopic activity. Clin. Rheumatol..

[8] D’haens G., Ferrante M., Vermeire S., Baert F., Noman M., Moortgat L., Geens P., Iwens D., Aerden I., Van Assche G. (2012). Fecal calprotectin is a surrogate marker for endoscopic lesions in inflammatory bowel disease. Inflamm. Bowel Dis..

[9] Rendek Z., Falk M., Grodzinsky E., Kechagias S., Hjortswang H. (2022). Oral omeprazole and diclofenac intake is associated with increased faecal calprotectin levels: A randomised open-label clinical trial. Eur. J. Gastroenterol. Hepatol..

[10] Fauny M., D’aMico F., Bonovas S., Netter P., Danese S., Loeuille D., Peyrin-Biroulet L. (2019). Faecal Calprotectin for the Diagnosis of Bowel Inflammation in Patients With Rheumatological Diseases: A Systematic Review. J. Crohn’s Colitis.

[11] Taylor W., Gladman D., Helliwell P., Marchesoni A., Mease P., Mielants H., CASPAR Study Group (2006). Classification criteria for psoriatic arthritis: Development of new criteria from a large international study. Arthritis Care Res..

[12] Rudwaleit M., van der Heijde D., Landewé R., Listing J., Akkoc N., Brandt J., Braun J., Chou C.T., Collantes-Estevez E., Dougados M. (2009). The development of Assessment of SpondyloArthritis international Society classification criteria for axial spondyloarthritis (part II): Validation and final selection. Ann. Rheum. Dis..

[13] Lai E.J., Calderwood A.H., Doros G., Fix O.K., Jacobson B.C. (2009). The Boston bowel preparation scale: A valid and reliable instrument for colonoscopy-oriented research. Gastrointest. Endosc..

[14] Fauny M., Moulin D., D’AMico F., Netter P., Petitpain N., Arnone D., Jouzeau J.-Y., Loeuille D., Peyrin-Biroulet L. (2020). Paradoxical gastrointestinal effects of interleukin-17 blockers. Ann. Rheum. Dis..

[15] Garrett S., Jenkinson T., Kennedy L.G., Whitelock H., Gaisford P., Calin A. (1994). A new approach to defining disease status in ankylosing spondylitis: The Bath Ankylosing Spondylitis Disease Activity Index. J. Rheumatol..

[16] Lukas C., Landewé R., Sieper J., Dougados M., Davis J., Braun J., van der Linden S., van der Heijde D. (2009). Development of an ASAS-endorsed disease activity score (ASDAS) in patients with ankylosing spondylitis. Ann. Rheum. Dis..

[17] Schoels M., Aletaha D., Funovits J., Kavanaugh A., Baker D., Smolen J.S. (2010). Application of the DAREA/DAPSA score for assessment of disease activity in psoriatic arthritis. Ann. Rheum. Dis..

[18] Daperno M., D’HAens G., Van Assche G., Baert F., Bulois P., Maunoury V., Sostegni R., Rocca R., Pera A., Gevers A. (2004). Development and validation of a new, simplified endoscopic activity score for Crohn’s disease: The SES-CD. Gastrointest. Endosc..

[19] Travis S.P.L., Schnell D., Krzeski P., Abreu M.T., Altman D.G., Colombel J.-F., Feagan B.G., Hanauer S.B., Lémann M., Lichtenstein G.R. (2011). Developing an instrument to assess the endoscopic severity of ulcerative colitis: The Ulcerative Colitis Endoscopic Index of Severity (UCEIS). Gut.

[20] Magro F., Sabino J., Rosini F., Tripathi M., Borralho P., Baldin P., Danese S., Driessen A., Gordon I.O., Iacucci M. (2022). ECCO Position on Harmonisation of Crohn’s Disease Mucosal Histopathology. J. Crohn’s Colitis.

[21] Gralnek I.M., Defranchis R., Seidman E., Leighton J.A., Legnani P., Lewis B.S. (2007). Development of a capsule endoscopy scoring index for small bowel mucosal inflammatory change. Aliment. Pharmacol. Ther..

[22] Tukey M., Pleskow D., Legnani P., Cheifetz A.S., Moss A.C. (2009). The utility of capsule endoscopy in patients With suspected Crohn’s disease. Am. J. Gastroenterol..

[23] Bergman M.J., Zueger P., Song J., Pivneva I., Betts K.A., Joshi A.D. (2018). Inflammatory Bowel Disease Is Associated with a Substantial Economic Burden in Patients with Psoriatic Arthritis and in Patients with Ankylosing Spondylitis. Arthritis & Rheumatol..

[24] Przepiera-Będzak H., Fischer K., Brzosko M. (2021). Axial spondyloarthritis and inflammatory bowel disease: Association between disease activity and endothelial dysfunction markers. Rheumatol. Int..

[25] Sanchez-Bilbao L., Martinez-Lopez D., Palmou-Fontana N., Armesto S., González-Gay M.A., Blanco R. (2020). Ab0829 Inflammatory Bowel Disease in Psoriatic Arthritis. Study of 306 Patients from a Single Universitary Center. Prevalence, Clinical Features and Relationship to Biologic Therapy. Ann. Rheum. Dis..

[26] Jadon D.R., Sengupta R., Nightingale A., Lindsay M., Korendowych E., Robinson G., Jobling A., Shaddick G., Bi J., Winchester R. (2017). Axial Disease in Psoriatic Arthritis study: Defining the clinical and radiographic phenotype of psoriatic spondyloarthritis. Ann. Rheum. Dis..

[27] Rudwaleit M., Haibel H., Baraliakos X., Listing J., Märker-Hermann E., Zeidler H., Braun J., Sieper J. (2009). The early disease stage in axial spondylarthritis: Results from the German Spondyloarthritis Inception Cohort. Arthritis Rheum..

[28] Baraliakos X., Braun J. (2015). Non-radiographic axial spondyloarthritis and ankylosing spondylitis: What are the similarities and differences?. RMD Open.

[29] Kopylov U., Starr M., Watts C., Dionne S., Girardin M., Seidman E.G. (2018). Detection of Crohn Disease in Patients with Spondyloar-thropathy: The SpACE Capsule Study. J. Rheumatol..

[30] Leirisalo-Repo M., Turunen U., Stenman S., Helenius P., Seppälä K. (1994). High frequency of silent inflammatory bowel disease in spondylarthropathy. Arthritis Rheum..

[31] De Vos M., Mielants H., Cuvelier C., Elewaut A., Veys E. (1996). Long-term evolution of gut inflammation in patients with spondy-loarthropathy. Gastroenterology.

[32] de Winter J.J., van Mens L.J., van der Heijde D., Landewé R., Baeten D.L. (2016). Prevalence of peripheral and extra-articular disease in ankylosing spondylitis versus non-radiographic axial spondyloarthritis: A meta-analysis. Arthritis Res. Ther..

[33] Stolwijk C., Essers I., van Tubergen A., Boonen A., Bazelier M.T., De Bruin M.L., de Vries F. (2015). The epidemiology of extra-articular manifestations in ankylosing spondylitis: A population-based matched cohort study. Ann. Rheum. Dis..

[34] Jayasooriya N., Baillie S., Blackwell J., Bottle A., Petersen I., Creese H., Saxena S., Pollok R.C., POP-IBD Study Group (2023). Systematic review with meta-analysis: Time to diagnosis and the impact of delayed diagnosis on clinical outcomes in inflammatory bowel disease. Aliment. Pharmacol. Ther..

[35] Mow W.S., Lo S.K., Targan S.R., Dubinsky M.C., Treyzon L., Abreu-Martin M.T., Papadakis K.A., Vasiliauskas E.A. (2004). Initial experience with wireless capsule enteroscopy in the diagnosis and management of inflammatory bowel disease. Clin. Gastroenterol. Hepatol..

[36] Østgård R., Deleuran B., Dam M., Hansen I., Jurik A., Glerup H. (2017). Faecal calprotectin detects subclinical bowel inflammation and may predict treatment response in spondyloarthritis. Scand. J. Rheumatol..

[37] Olofsson T., Lindqvist E., Mogard E., Andréasson K., Marsal J., Geijer M., Kristensen L.E., Wallman J.K. (2019). Elevated faecal calprotectin is linked to worse disease status in axial spondyloarthritis: Results from the SPARTAKUS cohort. Rheumatology.

[38] Poullis A., Foster R., Mendall M.A., Shreeve D., Wiener K. (2003). Proton pump inhibitors are associated with elevation of faecal cal-protectin and may affect specificity. Eur. J. Gastroenterol. Hepatol..

