# Supplementary material for: Prevalence of Undiagnosed Inflammatory Bowel Disease in Spondyloarthritis Patients
Source: J Clin Med. 2025 Jun 27;14(13):4569. doi: 10.3390/jcm14134569 (PMC12250012; doi:10.3390/jcm14134569)

Supplementary material

Supplementary Table S1. Criteria for the screening of inflammatory bowel disease in patients with spondyloarthritis [6].

Type of criteria	Criteria
Major	• Rectal bleeding<br>• Organic chronic diarrhea (onset more than 4 weeks before)<br>• Perianal disease
Minor	• Chronic abdominal pain (onset more than 4 weeks before; persistent or recurring).<br>• Iron-deficiency anemia or iron deficiency.<br>• Extraintestinal manifestations<br>• Fever or feverishness, no focality.<br>• Unexplained weight loss.<br>• Family history of inflammatory bowel disease.

Supplementary Table S2. Summary of findings in the colonoscopy and video capsule endoscopy in patients with IBD diagnosis.

**Colonoscopy (n=28)**	**N (%)**
Erythema	6 (21.4%)
Erosions/aphthous ulcers	19 (67.9%)
Superficial ulcers	8 (28.6%)
Deep ulcers	0
Strictures	1(3.6%)
**Video Capsule endoscopy (n=12)**	
Erythema	3 (25%)
Erosions/aphthous ulcers	11(91.6%)
Ulcers	1 (8.3)
Strictures	0

Supplementary Table S3. Bivariate analysis of factors associated with a diagnosis of IBD.

	IBD diagnosis		p-value	OR ($CI_{95\%}$)
	**Yes (N = 28)**	**No (N = 466)**		
Type of SpA				
PsA (Ref·)	6 (21.4%)	240 (51.5%)		
ax-SpA	22 (78.6%)	226 (48.5%)	0.002	3.89 (1.55-9.78)
Sex (male), n (%)	18 (64.3%)	229 (49.1%)	0.172	1.86 (0.84-4.12)
Age (years), mean ± SD	51.9 ± 12.7	51.5 ± 12.6	0.884	1.01 (0.97-1.03)
Age, n (%)			0.560	
< 45 (Ref·)	6 (21.4%)	138 (29.6%)		
45 to 65	18 (64.3%)	252 (54.1%)		1.64 (0.64-4.23)
≥ 65	4 (14.3%)	76 (16.3%)		1.21 (0.33-4.42)
IBD compatibles symptoms, n (%)	7 (25.0%)	50 (10.7%)	0.032	2.77 (1.12-6.85)
Number of symptoms associated with IBD, n (%)			0.062	
0 (Ref·)	21 (75.0%)	416 (89.3%)		
1	2 (7.1%)	18 (3.9%)		2·20 (0.48-10.12)
≥2	5 (17.9%)	32 (6.9%)		3·09 (1.09-8.75)
Findings associated with IBD, n (%)				
Rectal bleeding	0 (0%)	9 (1.9%)	0.988	-
Organic chronic diarrhea	2 (7.1%)	10 (2.1%)	0.144	3.51 (0.73-16.84)
Chronic abdominal pain	3 (10.7%)	14 (3.0%)	0.030	3.87 (1.05-14.36)
Extraintestinal manifestations	2 (7.1%)	13 (2.8%)	0.206	2.68 (0.57-12.51)
Perianal disease	1 (3.6%)	8 (1.7%)	0.411	2.12(0.26-17.57)
Iron-deficiency anemia	1 (3.6%)	9 (1.9%)	0.445	1.88 (0.23-15.39)
Weight loss	0 (0%)	5 (1.1%)	0.746	-
Vitamin B12 deficiency	4 (14.3%)	22 (4.7%)	0.028	3.36 (1.07-10.54)
FC (µg/g), median (IQR)	412 (179; 902)	45 (30; 144)	<0.001	
Calprotectin, n (%)				
< 80 µg/g (Ref·)	2 (7.1%)	318 (68.2%)		
≥ 80 µg/g	26 (92.9%)	148 (31.8%)	<0.001	27.93 (6.54-119.2)
HLAB27 positive, n (%)	20/24 (83.3%)	187/351 (53.3%)	0.004	4.39 (1.47-13.09)

axSpA: axial spondylarthritis; IBD: inflammatory bowel disease; IQR: interquartile range; PsA: psoriatic arthritis; SpA: spondylarthritis

Supplementary Table S4. Symptoms consistent with IBD by type of SpA.

	PsA (N = 246)	axSpA (N = 248)	p-value	Total (N = 494)
Symptoms consistent with IBD, n (%), n (%)			0.828	
0	219 (89.0%)	218 (88.0%)		437 (88.5%)
1	9 (3.7%)	11 (4.4%)		20 (4.1%)
2	8 (3.3%)	11 (4.4%)		19 (3.8%)
≥3	10 (4.0%)	8 (3.2%)		18 (3.6%)
Type of symptoms consistent with IBD, n (%)				
Rectal bleeding	6 (2.4%)	3 (1.2%)	0.493	9 (1.8)
Organic chronic diarrhea	6 (2.4%)	6 (2.4)	0.989	12 (2.4)
Chronic abdominal pain	8 (3.3%)	9 (3.6%)	0.818	17 (3.4)
Extraintestinal manifestations	4 (1.6%)	11 (4.4%)	0.069	15 (3.0)
Perianal disease	6 (2.4%)	3 (1.2%)	0.493	9 (1.8)
Iron-deficiency anemia	6 (2.4%)	4 (1.6%)	0.740	10 (2.0)
Weight loss	3 (1.2%)	2 (0.8)	0.993	5 (1.0)
Asthenia	5 (2.0)	11 (4.4)	0.210	16 (3.2)
Vitamin B12 deficiency	14 (5.7%)	12 (4.8)	0.692	26 (5.3)
Diagnosed with IBD, n (%)	6 (2.4)	22 (8.9)	<0.001	28 (5.7)

axSpA: axial spondylarthritis; IBD: inflammatory bowel disease; PsA: psoriatic arthritis; SpA: spondylarthritis.

Supplementary Figure S1. Flowchart according to basal determination of fecal calprotectin, use or not of NSAID, and presence or not of symptoms associated with IBD. NSAID: Nonsteroidal anti-inflammatory drug; Fc: Fecal calprotectin; IBD: Inflammatory bowel disease; VCE: Video capsule endoscopy; Dx: Diagnosis.

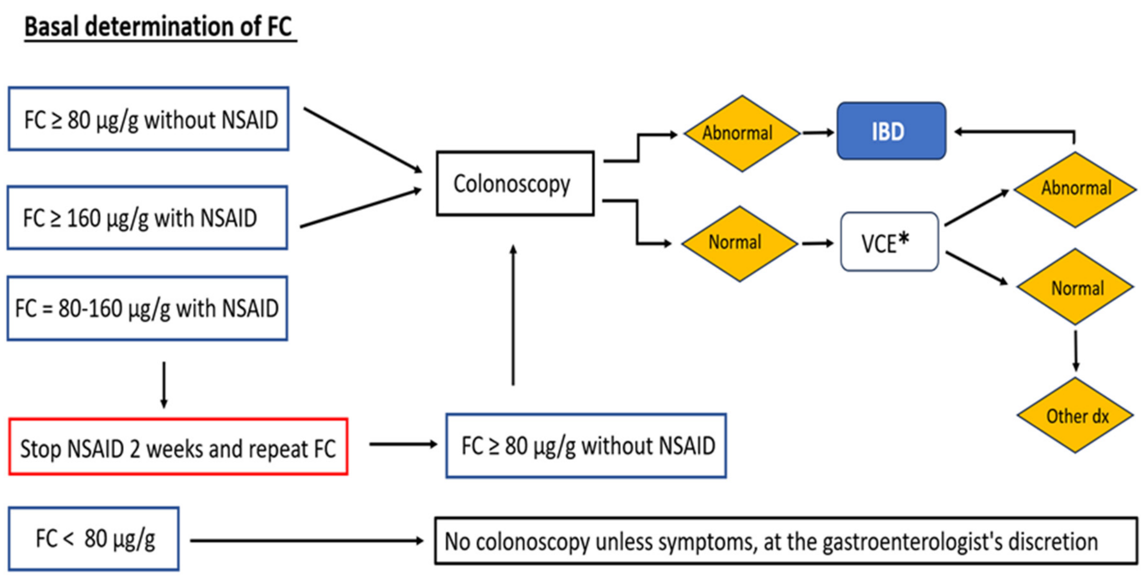

* If capsule endoscopy was contraindicated, the patient underwent a magnetic resonance enterography

NSAID: Non-steroidal anti-inflammatory drug; FC: fecal calprotectin; IBD: Inflammatory Bowel Disease; VCE: Video Capsule Endoscopy; Dx: Diagnosis.

Supplementary Figure S2. Receiver operating characteristic curve (ROC) for prediction of IBD based on basal FC in the evaluable population for IBD, excluding A) NSAID, B) PPI and C) NSAID + PPI taking patients.

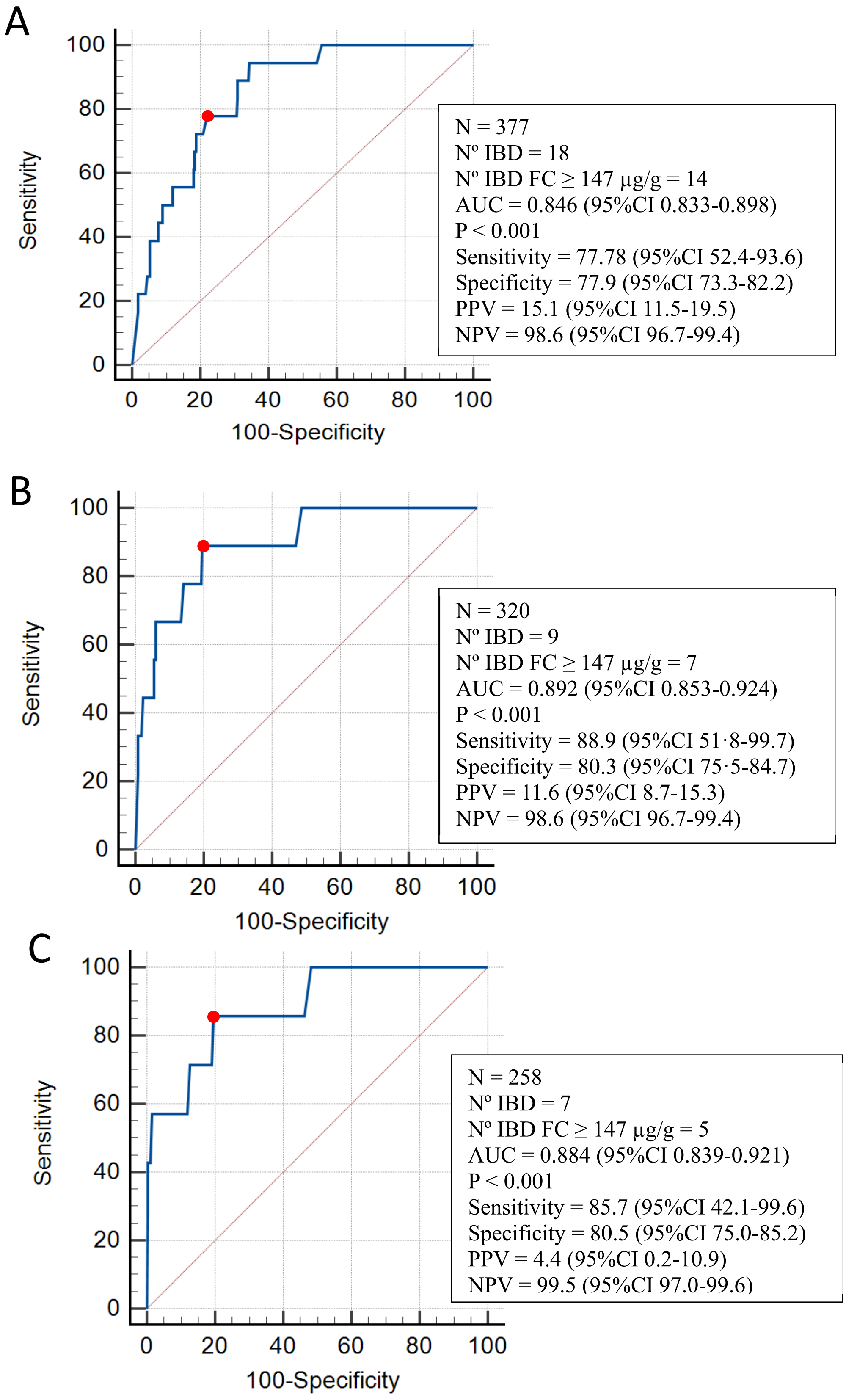

Supplement: Supplementary file 1 [file jcm-14-04569-s001.zip › jcm-3661218-supplementary.pdf]
